# Estimation of Heat-Attributable Mortality Using the Cross-Validated Best Temperature Metric in Switzerland and South Korea

**DOI:** 10.3390/ijerph18126413

**Published:** 2021-06-13

**Authors:** Jae Young Lee, Martin Röösli, Martina S. Ragettli

**Affiliations:** 1 Environmental and Safety Engineering Department, Ajou University, Suwon 16499, Korea; 2 Swiss Tropical and Public Health Institute, 4051 Basel, Switzerland; martina.ragettli@swisstph.ch (M.R.); martin.roosli@swisstph.ch (M.S.R.); 3 University of Basel, 4001 Basel, Switzerland

**Keywords:** temperature-mortality association, DLNM, cross validation

## Abstract

This study presents a novel method for estimating the heat-attributable fractions (HAF) based on the cross-validated best temperature metric. We analyzed the association of eight temperature metrics (mean, maximum, minimum temperature, maximum temperature during daytime, minimum temperature during nighttime, and mean, maximum, and minimum apparent temperature) with mortality and performed the cross-validation method to select the best model in selected cities of Switzerland and South Korea from May to September of 1995–2015. It was observed that HAF estimated using different metrics varied by 2.69–4.09% in eight cities of Switzerland and by 0.61–0.90% in six cities of South Korea. Based on the cross-validation method, mean temperature was estimated to be the best metric, and it revealed that the HAF of Switzerland and South Korea were 3.29% and 0.72%, respectively. Furthermore, estimates of HAF were improved by selecting the best city-specific model for each city, that is, 3.34% for Switzerland and 0.78% for South Korea. To the best of our knowledge, this study is the first to observe the uncertainty of HAF estimation originated from the selection of temperature metric and to present the HAF estimation based on the cross-validation method.

## 1. Introduction

Excessive heat exposure is a well-known public health problem. Several studies have examined the association between daily temperature and mortality based on historical data [1,2,3,4,5,6,7,8,9,10,11,12,13,14,15,16,17]. Among various temperature indices, the best predictor of heat-related mortality has been questioned and studied [18,19,20,21,22]. The results from previous studies revealed that the best model varies according to the study location. Barnett et al. (2010) argued that in 107 US cities, various temperature indices have the same predictive ability based on cross-validated residual [18]. Hajat et al. (2006) showed that in three European cities (London, Budapest, and Milan), the daily mean temperature was a better predictor of mortality than the daily maximum or daily minimum temperature because it characterized the complete profile of daily exposure [19]. Metzger et al. (2009) revealed that the maximum apparent temperature was better at predicting heat-related mortality in New York City, as compared to the daily mean, minimum, or maximum temperature based on the deviance explained metric [22].

Although it is unclear which temperature metric is the best, a majority of heat exposure studies have used the daily mean temperature as the temperature exposure metric to capture the overall daily temperature characteristics [1,2,3,4,5,6,7,8,9,10]; the second most popular choice of temperature metric is the daily maximum temperature [11,12,13,14]. The majority of these studies did not compare the models with multiple temperature metrics for more accurate estimation of the health impact.

This study aims to quantify the variability of the models with various temperature metrics and to suggest predicting heat-related mortality based on the cross-validated best model. To the best of our knowledge, no previous studies focused on this uncertainty in heat-related mortality estimation. To this end, this study modeled the relationship of temperature and mortality in selected cities of Switzerland and South Korea, based on eight temperature metrics (mean, maximum, minimum temperature, maximum temperature in daytime, minimum temperature in nighttime, mean, maximum, and minimum apparent temperature). Among these models, the best one was selected via cross-validation method, and the heat-related mortality was estimated using the best model. It is assumed that the analyses of two countries with different summer climates (hot and dry summers in Switzerland, and hot and humid summers in South Korea) are informative, such that the results can be generalized.

## 2. Method

### 2.1. Data Collection

Historical data on daily mortality, including non-external causes (ICD-10 A00-R99) and accidents (ICD-10 V01-X59), were obtained. Moreover, eight daily temperature metrics for eight cities in Switzerland (Basel, Bern, Geneva, Lausanne, Lugano, Lucerne, St. Gallen, and Zurich) and six cities in South Korea (Busan, Daegu, Daejeon, Gwangju, Incheon, and Seoul) were collected. Table 1 presents the descriptions and sources of the data. Figure S1 shows the map of the study locations. Daily temperature data on several meteorological indicators from a representative monitoring station in each city were collected from the IDAweb (Federal Office of Meteorology and Climatology Switzerland, MeteoSwiss) and the Korea Meteorological Administration. It included daily mean temperature (tmean), daily maximum temperature (tmax), daily minimum temperature (tmin), daytime maximum temperature (tmax_day), and nighttime minimum temperature (tmin_night). To determine the combined effect of heat and humidity, the daily mean, maximum, and minimum apparent temperatures (tmean_app, tmax_app, and tmin_app) were also assessed. The formula for apparent temperature is provided in the Appendix A.

### 2.2. Temperature–Mortality Relationship Assessment

To assess the temperature–mortality relationship, the two-stage time-series analysis was used, as described in previous studies [23,24]. The distributed lag nonlinear model (DLNM) used in this study is presented in Equation (1):

(1) log(E[Mortality])=CB+DOW+NS(time)

Here, CB is a cross-basis modeling of a lagged nonlinear effect of temperature, DOW is the day of the week (to control for daily variation), and NS(time) is a natural cubic spline with four degrees of freedom per year modeling seasonal and long-term variation. Three internal knots were placed at the 10th, 75th, and 90th percentiles of regional temperature to model the nonlinearity of the temperature effect [2]. With respect to mortality, a quasi-Poisson distribution was assumed. A maximum lag of 10 days was modeled with two logarithmic, equally spaced internal knots to capture the delayed effects of heat and short-term harvesting [25].

After DLNM modeling, a meta-analysis of all city-specific models from each country was performed to obtain the pooled temperature–mortality relationship. Additionally, the best linear unbiased prediction was conducted, based on the pooled and modeled relationships [24]. The minimum mortality temperature (MMT), which is defined as the temperature at which the temperature-attributable mortality is the smallest, was calculated based on the method described in Tobias et al. (2017) [26]. Identical methodologies and models were used for both countries.

### 2.3. Assessment of the Best Predictor for Temperature-Related Mortality

To analyze the best predictor for temperature-related mortality among various temperature metrics, the cross-validation method was used to avoid overfitting. Among the baseline period (1995–2015) data, for each round of cross-validation, a particular year’s data was selected as a validation data set and the remaining data were chosen as training dataset. For example, 1995 was used for validation, and 1996–2015 was selected for training in the first round; moreover, 1996 was targeted for validation, and 1995 and 1997–2015 were chosen for training in the second round, and so on. Then, the temperature–mortality relationship was obtained based on DLNM using the training set (20 years, e.g., 1996–2015 for the first round) and evaluated the model on the validation set (1 year, e.g., 1995 for the first round). For the evaluation, the DLNM model was used to estimate the daily mortality during the validation period, and the R2 was calculated by comparing the estimated and measured daily mortality. This process was performed iteratively for each year of the baseline period. Among the various temperature metrics, the best predictor among the various temperature metrics, which gave the maximum overall R2 throughout 1995–2015, was considered.

### 2.4. Heat-Attributable Fraction

Equation (2) shows the heat-attributable fraction (HAF), which is the ratio of heat-attributable mortality (the numerator) to the total mortality (the denominator).

(2) $$\mathrm{HAF}=\frac{\sum_{i\in B}m_{i}\cdot \left(1-\frac{1}{{\mathrm{RR}}_{i}}\right)}{\sum_{j\in A}m_{j}},\;B=\left\{i\in A|T_{i}\geq \mathrm{MMT}\right\}$$

Here, A is a set whose elements are the days in the study period; B is the subset of A, whose elements are the days when the temperature is above the MMT; mi is the daily mortality for day i; T_i_ is the temperature for day i, RR_i_ which stands for the relative risk is the ratio of mortality increase when exposed to temperature T_i_, and m_j_ is the daily mortality for day j. The term 1-1/RR_i_ in the numerator is the daily heat-attributable risk which is identical to the definition in [9].

HAF estimation was performed for each temperature metric for comparison. Similarly, the extreme-heat-attributable fraction (EHAF) and moderate-heat-attributable fraction (MHAF) are defined as follows:

(3) $$\mathrm{EHAF}=\frac{\sum_{i\in C}m_{i}\cdot \left(1-\frac{1}{{\mathrm{RR}}_{i}}\right)}{\sum_{j\in A}m_{j}},\;C=\left\{i\in A|T_{i}\geq P_{90}\right\}$$

(4) $$\mathrm{MHAF}=\frac{\sum_{i\in D}m_{i}\cdot \left(1-\frac{1}{{\mathrm{RR}}_{i}}\right)}{\sum_{j\in A}m_{j}},\;D=\left\{i\in A|\mathrm{MMT}\leq T_{i}<P_{90}\right\}$$

Here, P_90_ is the 90th temperature percentile.

## 3. Results

**Figure 1:**
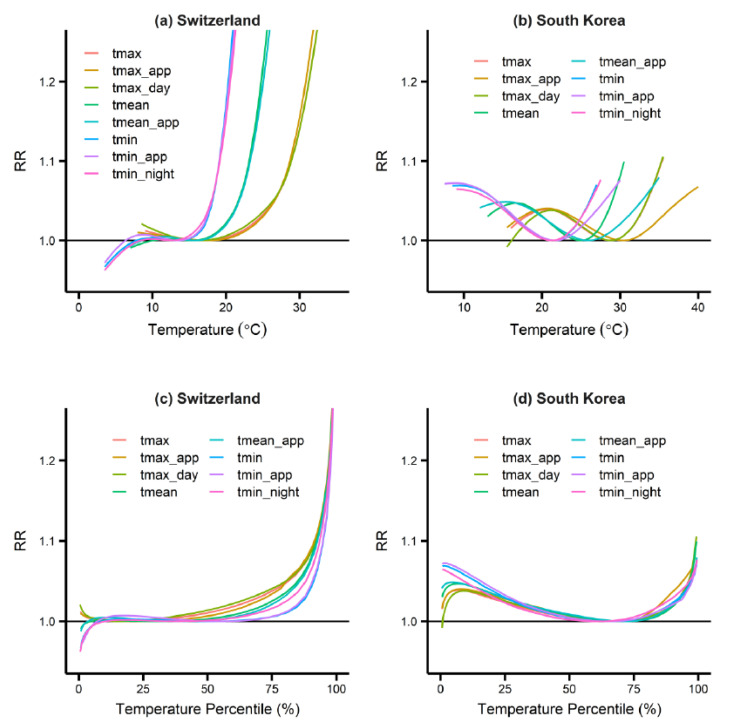
Temperature–mortality relationship for various temperature measures in (**a**) Switzerland and (**b**) South Korea; temperature percentile–mortality relationship for various temperature measures in (**c**) Switzerland and (**d**) South Korea. The curves are shown for the temperature range between 0.5 and 99.5 percentiles. Exposure–response associations are reported as relative risks (RR) for a cumulative 10-d lag of warm-season temperature, versus the optimum temperature (corresponding to the temperature of minimum mortality).

Based on the DLNM models, the relationship between temperature and mortality was established for various temperature measures in Switzerland and South Korea (see Figure 1 for the relationship curves; see Supplementary Figure S2 (Switzerland) and S3 (South Korea) for a 95% confidence interval). The mortality increase was presented in RR (the relative risk) in *y*-axis, which is the ratio of the increased mortality to the mortality at the minimum mortality temperature (MMT) [26]. Figure 1a,b shows that the relationship curves are different from the measured values because of different temperature profiles (see Supplementary Table S1 for descriptive statistics). However, when they are shown based on the temperature percentile, as in Figure 1c,d, the curves among various temperature measures appear to be similar. This is because of the high correlation between the temperature measures (see Supplementary Table S2 for the correlation coefficients).

Despite the high correlation and similarity shown in Figure 1, the curves of the various temperature metrics show some differences. First, the variability in the minimum mortality percentile (MMP), which is defined as the temperature percentile at which the temperature-attributable-mortality is the smallest, is significant (see Table 2). In Switzerland, MMP ranged between 10.0% (tmin_night) and 55.7% (tmin_app) for eight temperature metrics, while in South Korea, it ranged between 62.0% (tmin_night) and 72.1% (tmean_app). In addition, the HAF that was estimated based on each temperature metric demonstrated remarkable variability (see Table 3). In Switzerland, HAF ranged between 2.69% (tmin_app) and 4.09% (tmax_day), while in South Korea, its value was between 0.61% (tmin) and 0.90% (tmax_app). Table 3 shows the extreme-heat attributable fraction (EHAF) and MHAF. These fractions also showed variability, depending on the selection of temperature measures. In Switzerland, MHAF has a higher variation than EHAF, while in South Korea, the reverse is true.

To evaluate the quality of the models based on various temperature measures, a cross-validation was performed. Table 4 summarizes the R^2^ values of the daily mortality estimation for the total validation set for each temperature measure. Table S3 summarizes the root mean squared error (RMSE) for comparison. In Switzerland, R^2^ values for eight measures ranged from 14.38% (tmin_night) to 15.45% (tmean), while in South Korea, R^2^ values ranged from 27.71% (tmin_app) to 29.87% (tmean). The R^2^ values are low because temperature-attributable mortality accounts for only a small fraction of the total mortality, which includes all non-external causes and accidents (see Table 3 for HAF). Among the eight measures, tmean is the best measure for mortality in both Switzerland and South Korea based on cross-validation. The results also show that the relative humidity incorporated in the form of the apparent temperature plays an insignificant role in modeling heat-attributable mortality based on R^2^.

Given that each city is unique in terms of socio-economic and demographic aspects, the best model for each city tends to vary. We evaluated the relationships between temperature percentiles and mortality for various temperature measures for each city of Switzerland (Supplementary Figure S4) and South Korea (Supplementary Figure S5). Supplementary Table S4 presents city-specific cross-validation results. Based on the cross-validated R^2^ values, the best model varies. Moreover, no one measure is consistently better than the others. Tmean is ideal for modeling the relationship in Lucerne, Daegu, Incheon, and Seoul. Tmean_app, tmin_night, tmin, and tmax_app are the best in the two cities, while tmin_app and tmax_day are the best in one city. Supplementary Figures S6 and S7 highlight the best model curves in the cities of Switzerland and South Korea, respectively. These city-specific best models show higher R^2^ values (0.28%–1.28%) than the average R^2^ values for eight measures (see Supplementary Table S4). Using these city-specific best models, the overall R^2^ values between the measured and estimated daily mortality for the total study cities were 15.47% for Switzerland and 29.90% for South Korea. These are marginally better than the model that is based on tmean (see Table 4). In addition, based on the city-specific best models, the HAF is 3.34% in Switzerland and 0.78% in South Korea (see Table 3). These results are similar to the estimates obtained using tmean (3.29% in Switzerland and 0.72% in South Korea).

## 4. Discussion

The question about the best temperature metric to predict mortality has been addressed by previous researchers. However, the answer is dependent on the study location and the measure of goodness. Metzger et al. (2009) argued that the maximum apparent temperature is a better predictor of mortality in New York City than the daily mean, minimum, or maximum temperature, based on the explained deviance [22]. However, there was little evidence to support the argument for the cities of Switzerland and South Korea based on cross-validation. Barnett et al. (2010) [18] argued that there was no specific temperature metric that was superior to any other based on the cross-validated residual. Furthermore, they had the same predictive ability because of the high correlation between temperature metrics. Based on the results of the 14 cities selected for this study, it is argued that no measure is consistently better than the others. However, the argument about the same predictive ability has to be revisited, as models with different temperature metrics result in large variability (from 2.69% to 4.09% in Switzerland and from 0.61% to 0.90% in South Korea) when estimating the HAF. To better estimate the HAF, the cross-validation method is suggested to find the best temperature measure and to get an estimation based on the best measure. The cross-validation method can select the best temperature measures with a lower risk of overfitting than other commonly used methods, such as QAIC [27,28,29]. In our study, tmean was the best measure for both Switzerland and South Korea. Additionally, the city-specific best metric can be used in a multi-city study for better estimation; however, our result suggests that the improvement is marginal.

This study has a few limitations and drawbacks. Temperature was measured at an official station located in each city. It was assumed that the temperature data from the station could be applied as the temperature was exposed to individuals. Uncertainty of individual exposure is expected to result in the underestimation of the HAF. The R^2^ values of the best model used in this study are 15.47% for Switzerland and 29.90% for South Korea, which are quite low. This is because there are diverse causes of mortality, such as accidents, infections, diseases, and other environmental causes. For example, ambient ozone and particulate matters are known to have adverse health effects, thereby acting as confounding factors in modeling the temperature effects. Such confounding factors were not included in our DLNM model. Information on socio-economic and socio-demographic factors, such as proliferation of household air conditioning, insulation of buildings, frequency of outdoor activities, and demographic distributions was also not available. These factors may help explain city-specific differences and improve the quality of the model.

## 5. Conclusions

This study explored the relationship between temperature and mortality for eight temperature metrics in 14 cities of Switzerland and South Korea from May to September of 1995–2015. It was observed that the MMP and HAF for each metric was different. On evaluating the goodness of models based on cross-validation, it was revealed that tmean was the best measure for Switzerland and South Korea. However, there was no particular metric that was consistently better than the others. Therefore, to obtain a better estimation of MMP and HAF, the cross-validated best model (the overall best or the city-specific best) has been suggested.

## Figures and Tables

**Table 1:** Overall description of the data.

Country	Switzerland	South Korea
Year	1995–2015	1995–2015
Month	May-Sep	May-Sep
Cities	Basel, Bern, Geneva, Lausanne, Lugano, Lucerne, St. Gallen, Zurich	Busan, Daegu, Daejeon, Gwangju, Incheon, Seoul
Mortality causes (ICD-10)	A00-R99 (non-external) V01-X59 (accidents)	A00-R99 (non-external) V01-X59 (accidents)
Mortality data source	Federal Office of Statistics	Korea Bureau of Statistics
Temperature data source	MeteoSwiss, the Swiss Federal Office of Meteorology and Climatology	Korea Meteorological Administration

**Table 2:** Minimum mortality percentile (MMP) and minimum mortality temperature (MMT) of the temperature–mortality relationship using eight temperature metrics in Switzerland and South Korea.

	Switzerland		South Korea	
	MMP	MMT	MMP	MMT
tmean	31.4%	15.3 °C	70.9%	24.9 °C
tmax	17.7%	16.9 °C	66.2%	28.7 °C
tmax_day	15.3%	16.3 °C	67.3%	28.8 °C
tmin	50.3%	13.3 °C	65.9%	21.3 °C
tmin_night	10.0%	8.4 °C	62.0%	21.2 °C
tmean_app	39.8%	15.8 °C	72.1%	25.9 °C
tmax_app	25.6%	17.7 °C	68.9%	30.4 °C
tmin_app	55.7%	13.5 °C	65.5%	21.7 °C

**Table 3:** Heat-attributable fraction (HAF), extreme-heat-attributable fraction (EHAF), and moderate-heat-attributable fraction (MHAF) based on the various temperature metrics in Switzerland and South Korea. Average = the average of fractions from eight temperature metrics. City-specific best model = fractions calculated using the city-specific best models based on cross-validation.

	Switzerland			South Korea		
	HAF	EHAF	MHAF	HAF	EHAF	MHAF
tmean	3.29%	1.88%	1.41%	0.72%	0.63%	0.10%
tmax	3.94%	1.93%	2.01%	0.74%	0.61%	0.13%
tmax_day	4.09%	1.94%	2.16%	0.74%	0.61%	0.13%
tmin	2.76%	1.67%	1.10%	0.61%	0.47%	0.14%
tmin_night	3.31%	1.91%	1.41%	0.71%	0.51%	0.20%
tmean_app	3.08%	1.88%	1.20%	0.72%	0.60%	0.12%
tmax_app	3.90%	1.96%	1.94%	0.90%	0.63%	0.27%
tmin_app	2.69%	1.68%	1.01%	0.61%	0.47%	0.15%
Average	3.38%	1.86%	1.53%	0.72%	0.57%	0.15%
City-specific best model	3.34%	1.91%	1.43%	0.78%	0.67%	0.11%

**Table 4:** Cross-validated R2 values of DLNM models based on various temperature metrics in Scheme 2. values are calculated by comparing the measured and estimated daily mortality on the validation data set. Average = the average of R2 from eight temperature metrics. City-specific best model = R2 achieved using the city-specific best models based on the cross-validation.

	Switzerland	South Korea
tmean	15.45%	29.87%
tmax	15.09%	28.47%
tmax_day	14.89%	28.43%
tmin	14.71%	28.02%
tmin_night	14.38%	28.43%
tmean_app	15.31%	29.49%
tmax_app	15.15%	28.72%
tmin_app	14.43%	27.71%
Average	14.93%	28.64%
City-specific best model	15.47%	29.90%

## Data Availability

The datasets used and/or analyzed during the current study are available from the corresponding author on reasonable request.

## Supplementary Materials

The following are available online at https://www.mdpi.com/article/10.3390/ijerph18126413/s1. Figure S1: Map of the study locations. Figure S2: Temperature percentile-mortality relationships with 95% confidence interval in Switzerland. The curves were shown for the temperature range between 0.5 and 99.5 percentiles. The red dotted line shows the minimum mortality percentile (MMP). Figure S3: Temperature percentile-mortality relationships with 95% confidence interval in South Korea. The curves were shown for the temperature range between 0.5 and 99.5 percentiles. The red dotted line shows the minimum mortality percentile (MMP). Table S1: Descriptive statistics of temperature metrics in Switzerland and South Korea. Table S2: Correlation between various temperature metrics in Switzerland and South Korea. Table S3: Cross-validated root mean squared error (RMSE) values of DLNM models based on various temperature metrics in cities of Switzerland and South Korea. Table S4: Cross-validated R^2^ values of DLNM models based on various temperature metrics in cities of Switzerland and South Korea. The R^2^ values are between the measured and estimated daily mortality on the validation data set. Figure S4: Temperature percentile-mortality relationships in cities of Switzerland. The curves were shown for the temperature range between 0.5 and 99.5 percentiles. Figure S5: Temperature percentile–mortality relationships in cities of South Korea. The curves were shown for the temperature range between 0.5 and 99.5 percentiles. Figure S6: The city-specific best temperature percentile–mortality relationships in cities of Switzerland. The curves were shown for the temperature range between 0.5 and 99.5 percentiles. The grey curves were eight temperature percentile-mortality relationships shown in Figure S4. Figure S7: The city-specific best temperature percentile-mortality relationships in cities of South Korea. The curves were shown for the temperature range between 0.5 and 99.5 percentiles. The grey curves were eight temperature percentile–mortality relationships shown in Figure S5.

## Appendix A. Apparent Temperature Calculation

The apparent temperature, also known as the heat index, was developed by Steadman [30]. We used this index for our study as in Metzger et al. (2009). To calculate the apparent temperature, we used the Celsius version of the formula presented by Rothfusz [31], since it can well approximate the original tables by Steadman within ±0.7 °C and easy to calculate due to its polynomial form. The formula is given as below:

(A1) $$T_{\mathrm{app}}=c_{1}+c_{2}T+c_{3}R+c_{4}\mathrm{TR}+c_{5}T^{2}+c_{6}R^{2}+c_{7}T^{2}R+c_{8}{\mathrm{TR}}^{2}+c_{9}T^{2}R^{2}$$

Here, T_app_ is the apparent temperature in Celsius, T is the mean, maximum, or minimum temperature in Celsius, R is the relative humidity (percentage value between 0 and 100), c_1_ is −8.78469475556, c_2_ is 1.61139411, c_3_ is 2.33854883889, c_4_ is −0.14611605, c_5_ is −0.012308094, c_6_ is −0.0164248277778, c_7_ is 0.002211732, c_8_ is 0.00072546, and c_9_ is −0.000003582.
