# Supplementary material for: Estimation of Heat-Attributable Mortality Using the Cross-Validated Best Temperature Metric in Switzerland and South Korea
Source: Int J Environ Res Public Health. 2021 Jun 13;18(12):6413. doi: 10.3390/ijerph18126413 (PMC8296236; doi:10.3390/ijerph18126413)
Supplement: Supplementary file 1 [file ijerph-18-06413-s001.zip › ijerph-1232038-supplementary.pdf]

Estimation of heat-attributable mortality using the cross-validated best temperature metric in Switzerland and South Korea

Jae Young Lee[1,*], Martin Röösli[2,3], Martina S. Ragettli[2,3]

Affiliations:

[1] Environmental and Safety Engineering Department, Ajou University, Suwon, 16499, South Korea

[2] Swiss Tropical and Public Health Institute, 4051 Basel, Switzerland

[3] University of Basel, 4001 Basel, Switzerland

E-mail:

Jae Young Lee jaeylee@ajou.ac.kr

Martin Röösli martin.roosli@swisstph.ch

Martina S. Ragettli martina.ragettli@swisstph.ch

***Corresponding author**

Correspondence to Jae Young Lee

206, World cup-ro, Yeongtong-gu, Suwon, Gyeonggi-do 16499, South Korea

Tel: +82-31-219-2404

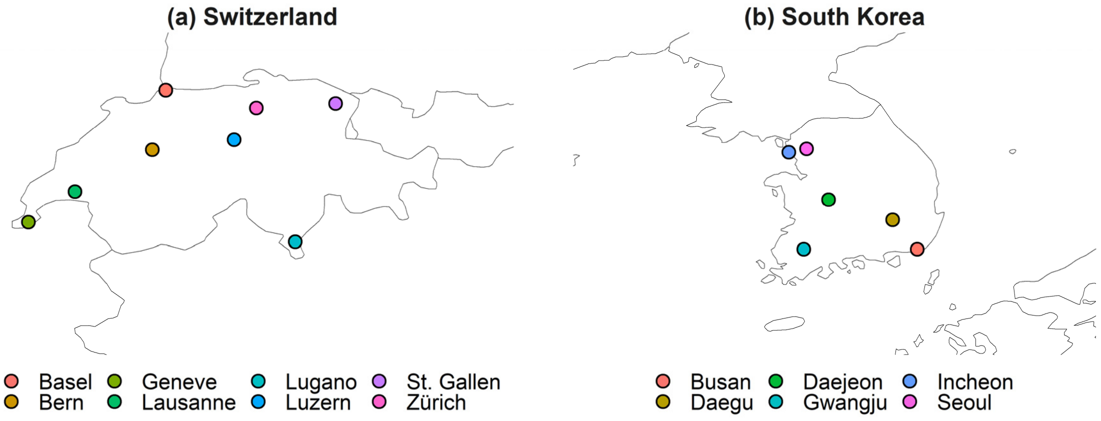

Fig. S1. Map of the study locations

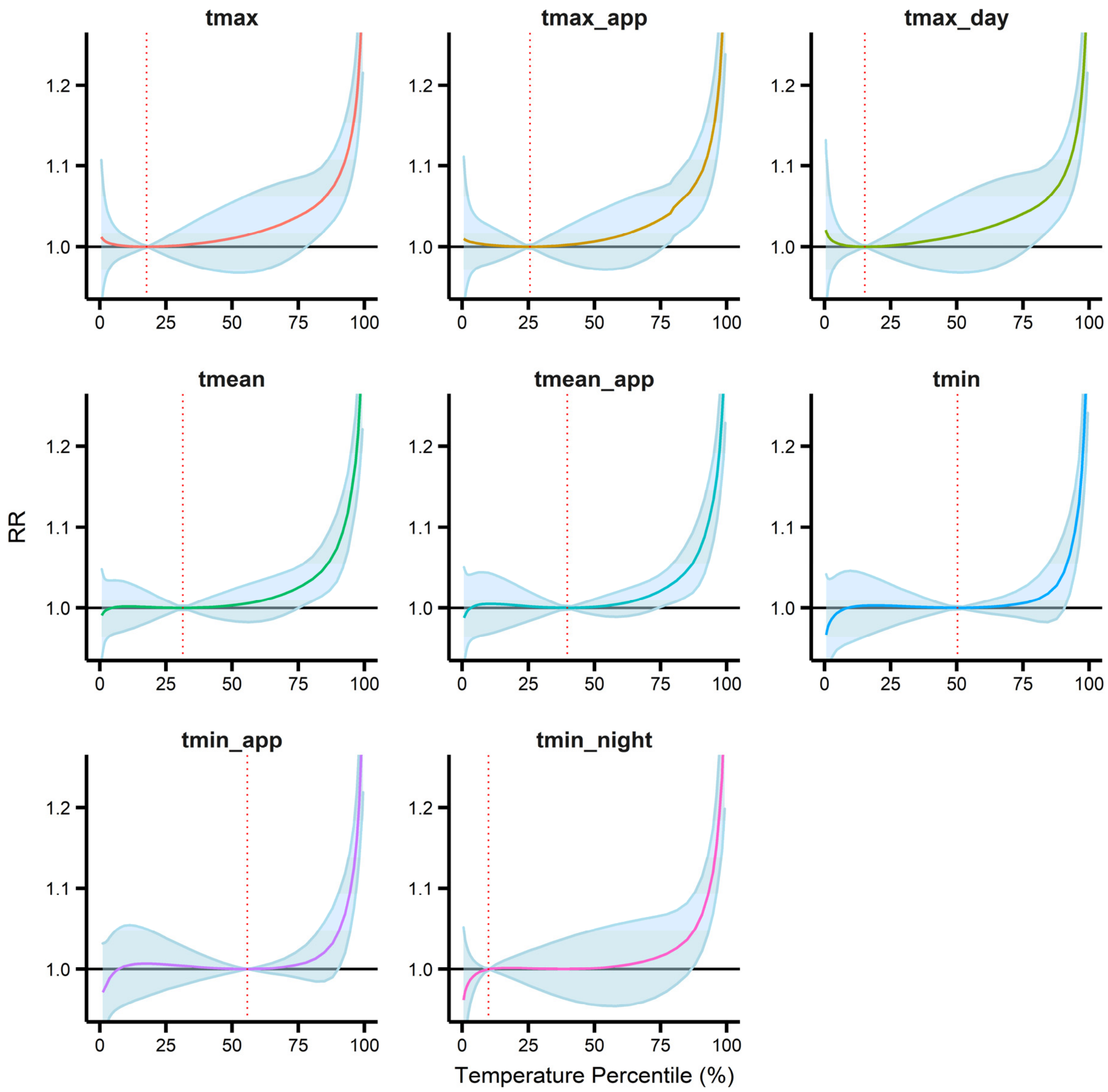

Fig. S2. Temperature percentile-mortality relationships with 95% confidence interval in Switzerland. The curves were shown for the temperature range between 0.5 and 99.5 percentiles. The red dotted line shows the minimum mortality percentile (MMP).

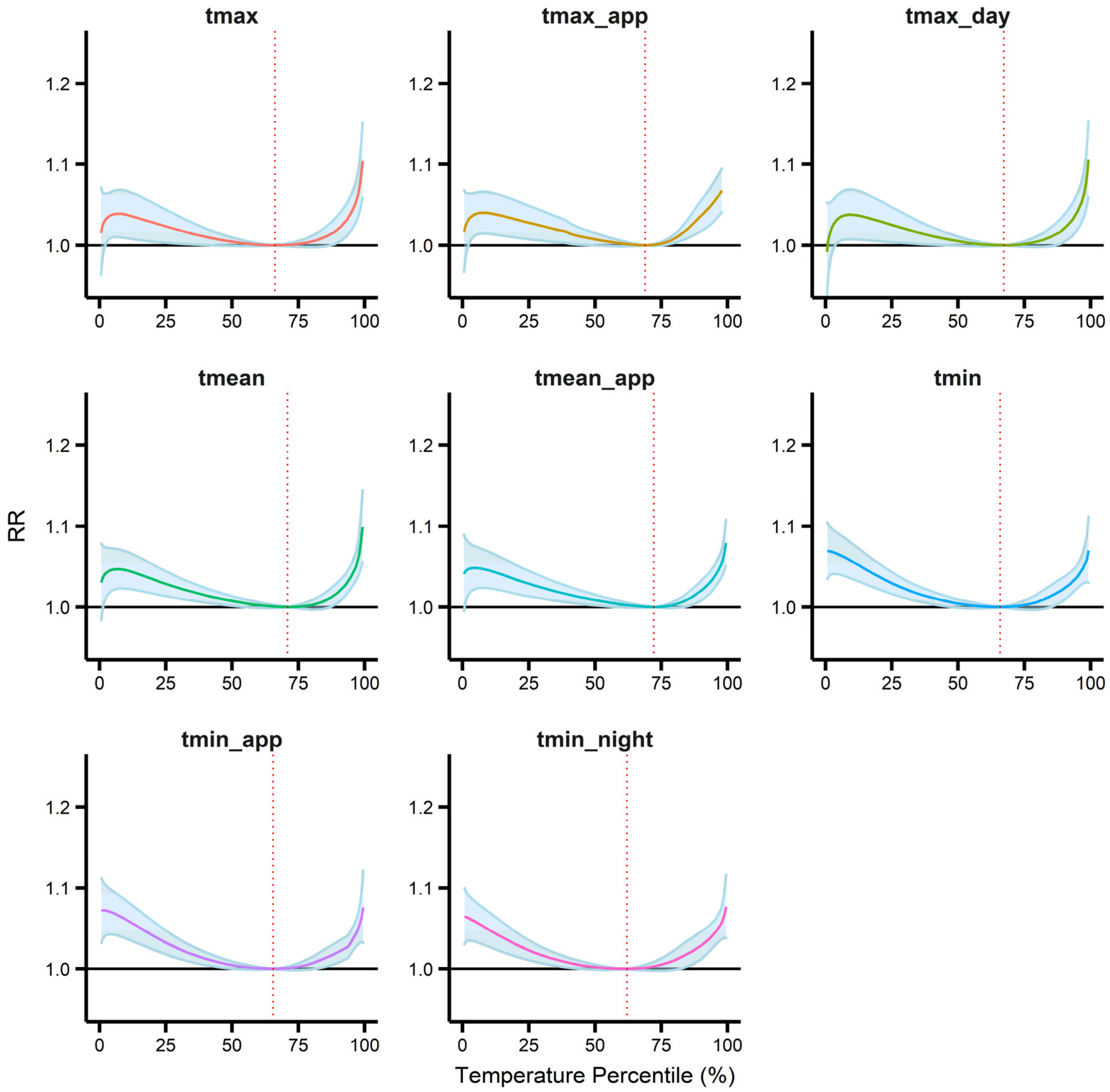

Fig. S3. Temperature percentile-mortality relationships with 95% confidence interval in South Korea. The curves were shown for the temperature range between 0.5 and 99.5 percentiles. The red dotted line shows the minimum mortality percentile (MMP).

Table S1. Descriptive statistics of temperature metrics in Switzerland and South Korea.

Country	Metric	Mean	Std.	p10	p25	p50	p75	p90
Switzerland	tmean	17.3	4.1	11.8	14.4	17.3	20.3	22.6
	tmax	21.8	5.1	15.0	18.2	21.9	25.5	28.3
	tmax_day	21.7	5.2	14.9	18.1	21.8	25.5	28.3
	tmin	13.1	3.8	8.0	10.5	13.2	15.8	17.9
	tmin_night	13.5	3.8	8.4	10.9	13.6	16.2	18.3
	tmean_app	16.9	4.4	11.1	13.9	17.0	20.1	22.6
	tmax_app	21.4	5.4	14.3	17.6	21.5	25.3	28.3
	tmin_app	12.7	4.1	7.1	9.9	12.8	15.6	17.9
South Korea	tmean	22.7	3.7	17.6	20.2	22.9	25.4	27.5
	tmax	26.8	4.0	21.5	24.1	27.0	29.6	31.8
	tmax_day	26.7	4.1	21.3	24.0	26.9	29.6	31.8
	tmin	19.2	4.2	13.2	16.3	19.6	22.4	24.6
	tmin_night	19.6	4.2	13.8	16.8	20.0	22.8	24.9
	tmean_app	23.3	4.8	17.2	20.1	23.2	26.4	30.0
	tmax_app	28.1	5.6	21.2	24.2	27.6	31.6	36.0
	tmin_app	19.4	4.8	12.7	16.1	19.8	22.9	25.3

Table S2. Correlation between various temperature metrics in Switzerland and South Korea.

Country	Metric	tmean	tmax	tmax_day	tmin	tmin_night	tmean_app	tmax_app	tmin_app
Switzerland	tmean	1.00	0.95	0.95	0.90	0.90	1.00	0.96	0.89
	tmax	0.95	1.00	1.00	0.75	0.80	0.95	1.00	0.74
	tmax_day	0.95	1.00	1.00	0.74	0.80	0.94	0.99	0.74
	tmin	0.90	0.75	0.74	1.00	0.88	0.91	0.77	1.00
	tmin_night	0.90	0.80	0.80	0.88	1.00	0.90	0.83	0.88
	tmean_app	1.00	0.95	0.94	0.91	0.90	1.00	0.96	0.91
	tmax_app	0.96	1.00	0.99	0.77	0.83	0.96	1.00	0.77
	tmin_app	0.89	0.74	0.74	1.00	0.88	0.91	0.77	1.00
South Korea	tmean	1.00	0.91	0.91	0.92	0.91	0.99	0.94	0.91
	tmax	0.91	1.00	1.00	0.70	0.74	0.88	0.95	0.69
	tmax_day	0.91	1.00	1.00	0.69	0.74	0.87	0.94	0.68
	tmin	0.92	0.70	0.69	1.00	0.92	0.93	0.80	1.00
	tmin_night	0.91	0.74	0.74	0.92	1.00	0.92	0.83	0.92
	tmean_app	0.99	0.88	0.87	0.93	0.92	1.00	0.95	0.93
	tmax_app	0.94	0.95	0.94	0.80	0.83	0.95	1.00	0.80
	tmin_app	0.91	0.69	0.68	1.00	0.92	0.93	0.80	1.00

Table S3. Cross-validated root mean squared error (RMSE) values of DLNM models based on various temperature metrics in cities of Switzerland and South Korea.

	Switzerland	South Korea
tmean	5.689	17.44
tmax	5.701	17.61
tmax_day	5.684	17.62
tmin	5.713	17.67
tmin_night	5.705	17.62
tmean_app	5.691	17.49
tmax_app	5.696	17.58
tmin_app	5.720	17.71
Average	5.700	17.59
City-specific best model	5.683	17.43

Table S4. Cross-validated R^2 values of DLNM models based on various temperature metrics in cities of Switzerland and South Korea. The R^2 values are between the measured and estimated daily mortality on the validation data set.

Model	Basel	Bern	Geneva	Lausanne	Lugano	Lucerne	St. Gallen	Zurich
tmean	2.76%	1.09%	0.58%	2.25%	1.67%	0.28%	0.15%	8.70%
tmax	2.56%	1.12%	0.43%	2.22%	1.53%	0.26%	0.17%	8.27%
tmax_day	2.57%	1.12%	0.39%	2.20%	1.51%	0.26%	0.18%	8.26%
tmin	2.82%	1.10%	0.17%	2.44%	1.53%	0.23%	0.21%	8.50%
tmin_night	2.88%	1.11%	0.19%	2.27%	1.51%	0.06%	0.10%	8.80%
tmean_app	2.62%	1.16%	0.58%	2.20%	1.68%	0.26%	0.14%	8.78%
tmax_app	2.46%	1.12%	0.55%	2.15%	1.72%	0.17%	0.13%	8.41%
tmin_app	2.73%	1.17%	0.17%	2.37%	1.43%	0.19%	0.17%	8.51%
Best model	tmin_night	tmin_app	tmean_app	tmin	tmax_app	tmean	tmin	tmin_night
Average R^2	2.68%	1.12%	0.38%	2.26%	1.57%	0.21%	0.16%	8.53%
Best R^2	2.88%	1.17%	0.58%	2.44%	1.72%	0.28%	0.21%	8.80%
Difference	0.21%	0.05%	0.20%	0.18%	0.15%	0.07%	0.05%	0.27%

Model	Busan	Daegu	Daejeon	Gwangju	Incheon	Seoul
tmean	8.40%	4.63%	4.93%	6.63%	17.21%	11.82%
tmax	7.70%	3.98%	4.86%	6.62%	16.90%	10.31%
tmax_day	7.72%	3.99%	4.85%	6.66%	16.97%	10.19%
tmin	8.19%	3.64%	4.64%	6.30%	16.93%	10.12%
tmin_night	7.95%	3.85%	4.67%	5.97%	16.81%	10.48%
tmean_app	8.42%	4.29%	4.92%	6.63%	16.87%	11.44%
tmax_app	7.95%	3.52%	5.30%	6.63%	16.51%	10.08%
tmin_app	8.02%	3.52%	4.65%	6.26%	16.84%	9.86%
Best model	tmean_app	tmean	tmax_app	tmax_day	tmean	tmean
Average R^2	8.04%	3.93%	4.85%	6.46%	16.88%	10.54%
Best R^2	8.42%	4.63%	5.30%	6.66%	17.21%	11.82%
Difference	0.38%	0.70%	0.45%	0.20%	0.33%	1.28%

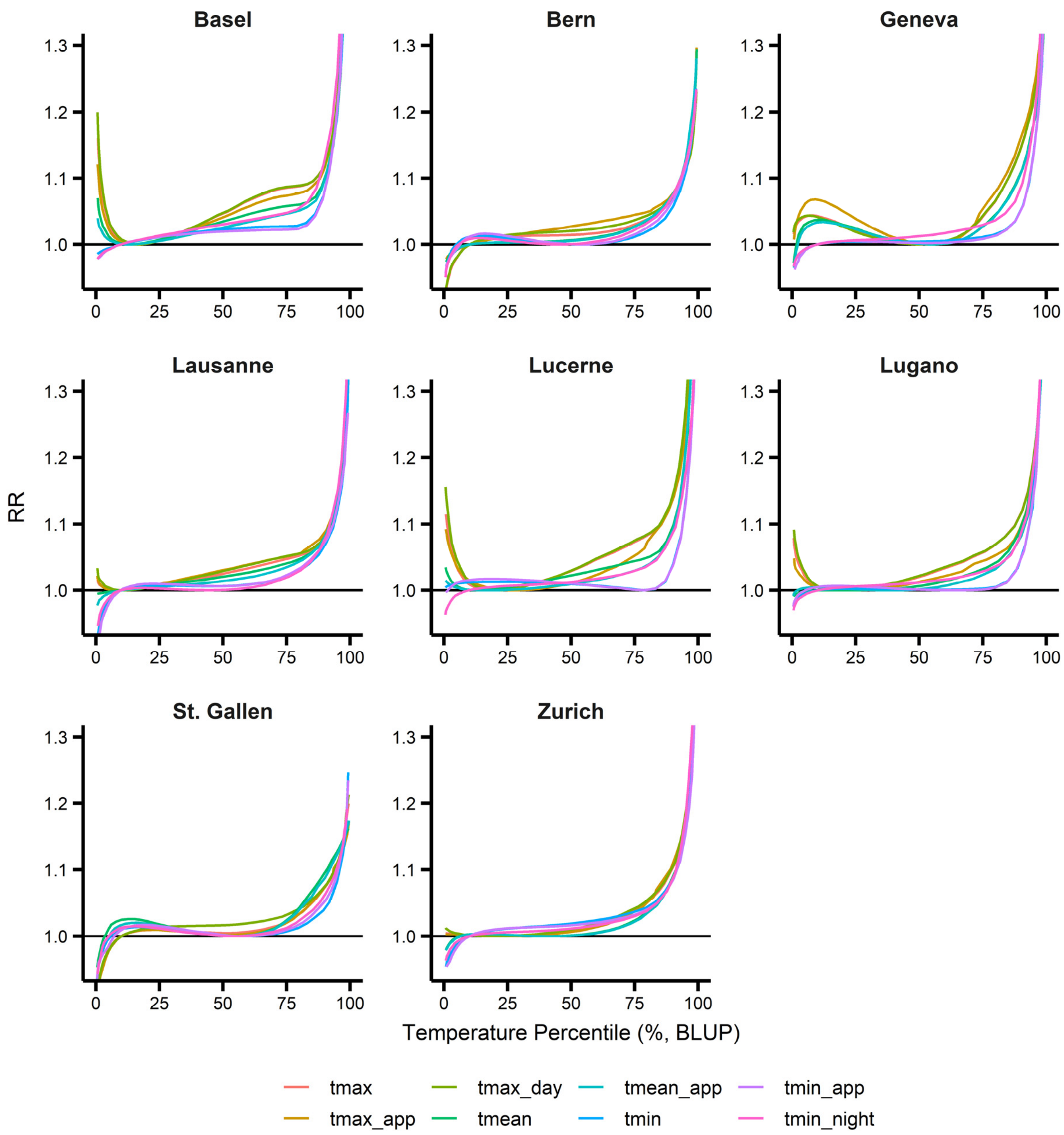

Fig. S4. Temperature percentile-mortality relationships in cities of Switzerland. The curves were shown for the temperature range between 0.5 and 99.5 percentiles.

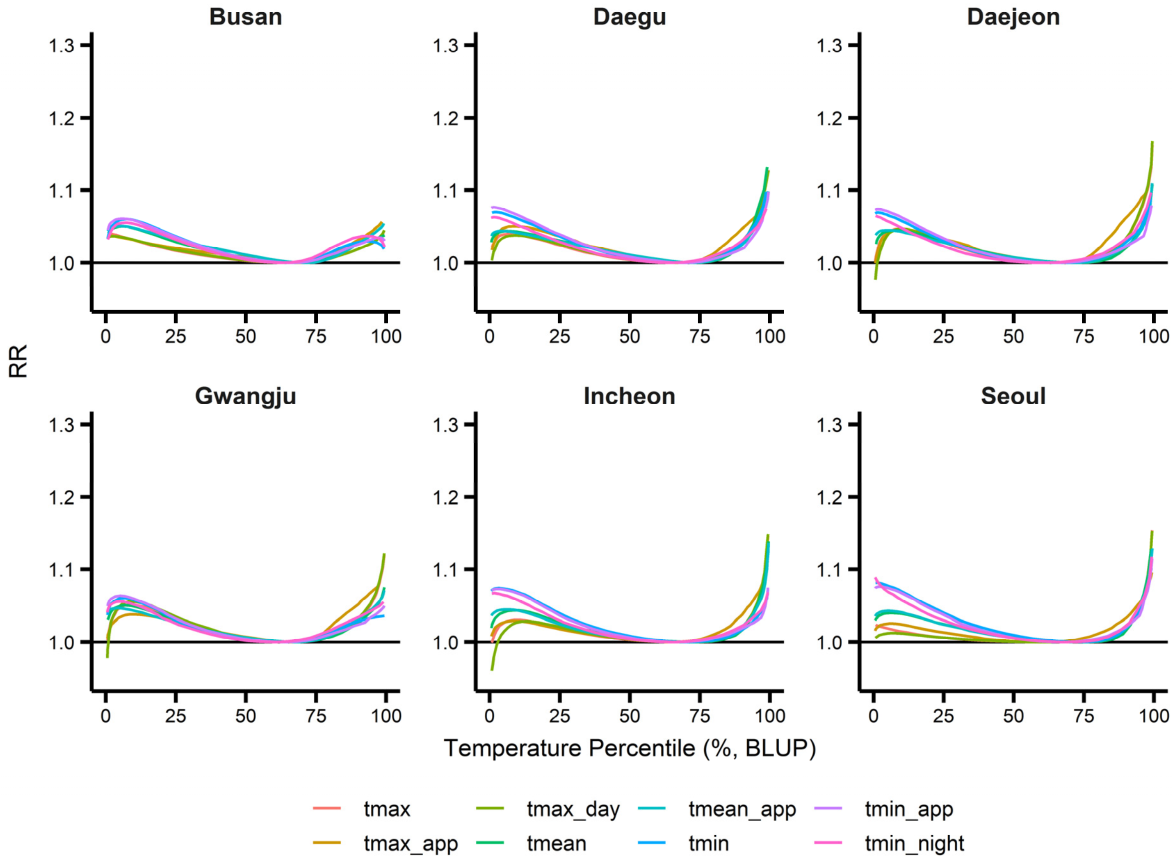

Fig. S5. Temperature percentile-mortality relationships in cities of South Korea. The curves were shown for the temperature range between 0.5 and 99.5 percentiles.

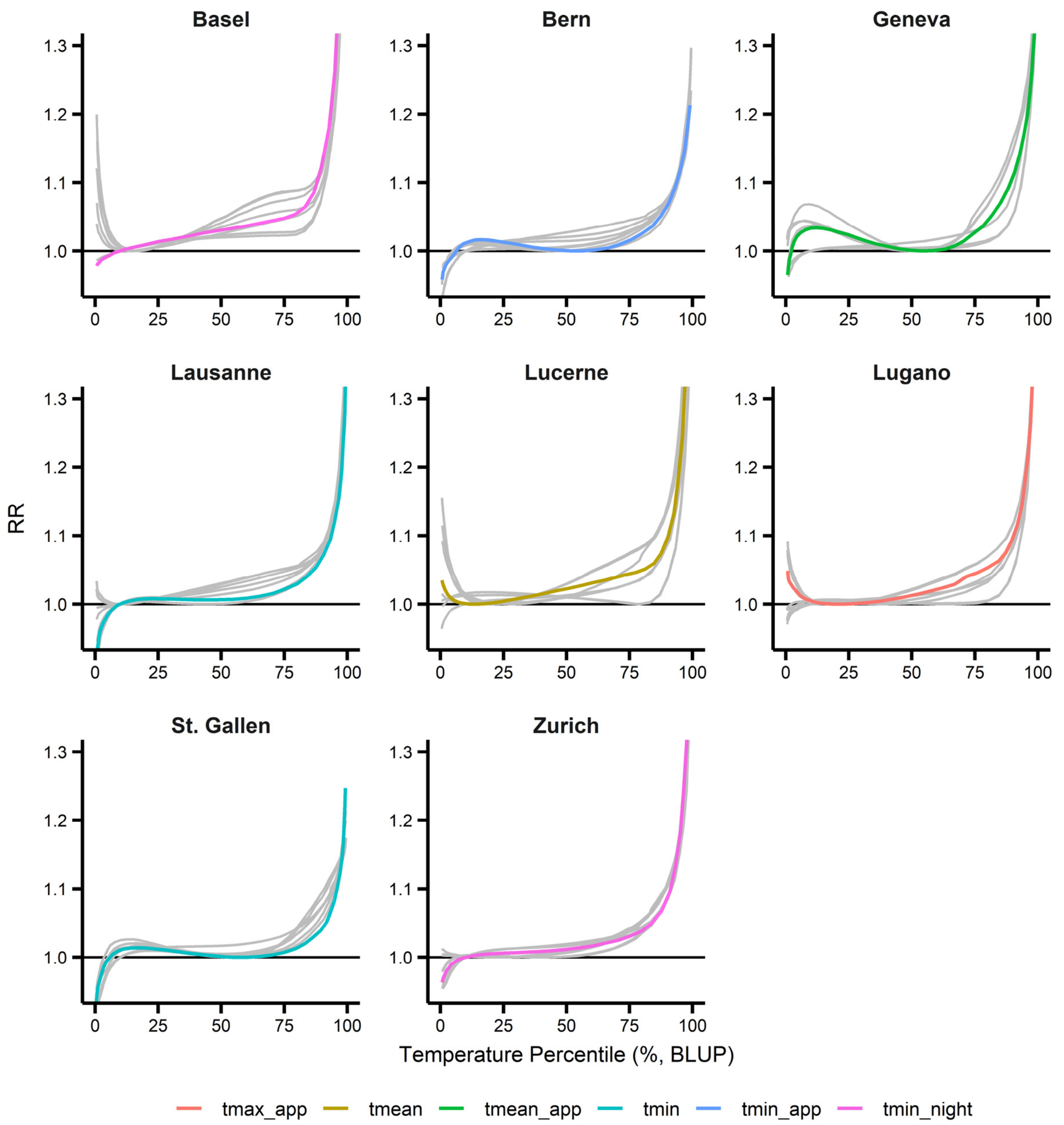

Fig. S6. The city-specific best temperature percentile-mortality relationships in cities of Switzerland. The curves were shown for the temperature range between 0.5 and 99.5 percentiles. The grey curves were eight temperature percentile-mortality relationships shown in Fig. S4.

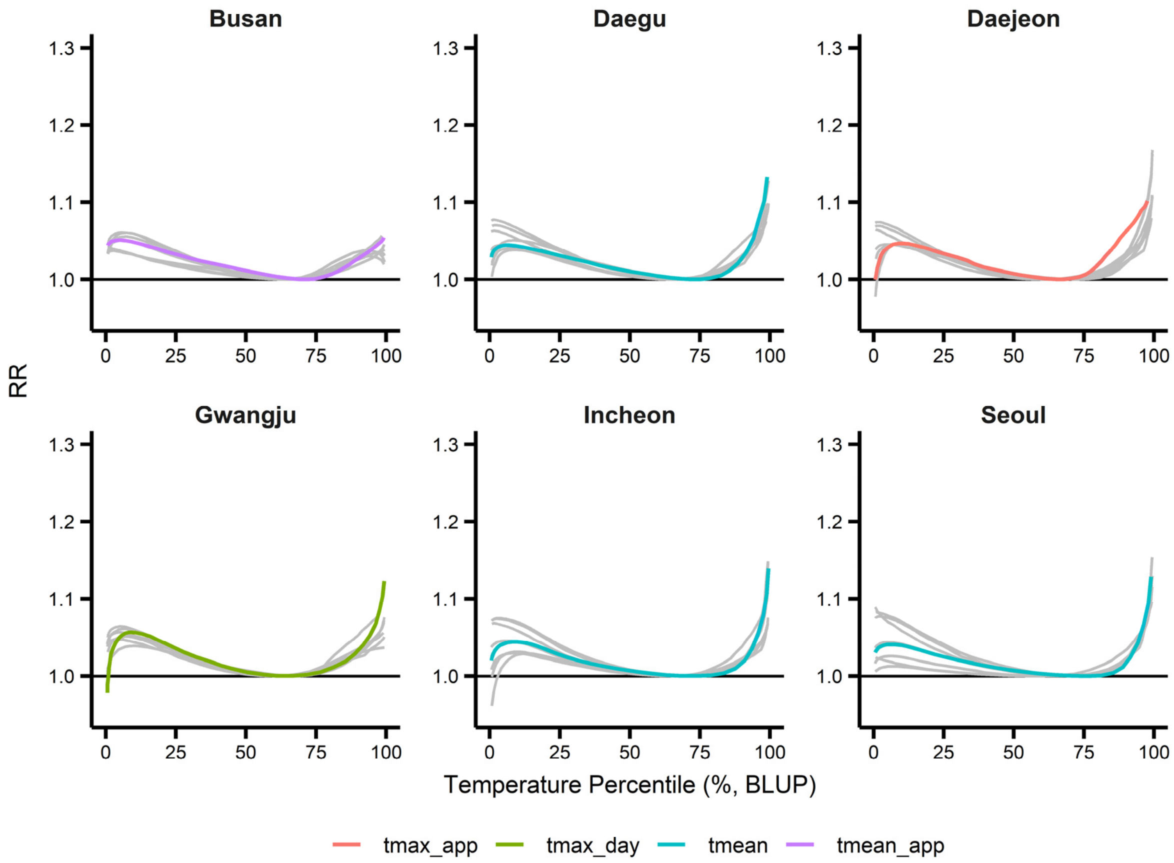

Fig. S7. The city-specific best temperature percentile-mortality relationships in cities of South Korea. The curves were shown for the temperature range between 0.5 and 99.5 percentiles. The grey curves were eight temperature percentile-mortality relationships shown in Fig. S5.
